# Microstructural integrity within the damaged region of the residual corticofugal projection from primary motor cortex predicts the effect of noninvasive neuromodulation targeting the spinal cord in chronic stroke

**DOI:** 10.1016/j.neurot.2025.e00607

**Published:** 2025-05-17

**Authors:** Michael A. Urbin, Fang Liu, Chan Hong Moon

**Affiliations:** a Human Engineering Research Laboratories, VA RR&D Center of Excellence, VA Pittsburgh Healthcare System, Pittsburgh, 15206, PA, USA; b Department of Physical Medicine & Rehabilitation, University of Pittsburgh, Pittsburgh, 15213, PA, USA; c Department of Radiology, University of Pittsburgh, Pittsburgh, 15213, PA, USA

**Keywords:** Stroke, Cerebrovascular accident, Corticospinal tract, Primary motor cortex, Neuromodulation

## Abstract

Distal limb impairment after neurological injury is largely a consequence of damage to descending tracts that structurally and functionally connect cortical motor areas with spinal motor neuron pools. Noninvasive neuromodulation strategies that aim to enhance cortico-spinal connectivity via spike timing-dependent mechanisms in the spinal cord rely on transmission of descending volleys across the residual tract. Whether variation in the aftereffects of noninvasive neuromodulation depends on the overall volume or microstructural integrity of fibers that survive injury is unknown. Here, paired corticospinal-motoneuronal stimulation (PCMS) was administered to increase cortico-spinal connectivity of the residual tract in humans with longstanding hand impairment due to stroke. Diffusion MRI was used to reconstruct the residual corticofugal projection from primary motor cortex. We found that fractional anisotropy of fibers within the region directly damaged by stroke accounted for 49.2 ​% of the variance in facilitation of motor-evoked potentials elicited by single-pulse transcranial magnetic stimulation. White matter volume within the damaged region was only weakly correlated with the observed change. Microstructure in caudal portions of the residual tract subject to secondary degeneration strongly predicted voluntary and stimulation-evoked activation of spinal motor neurons pools innervating the paretic hand but were unrelated to PCMS aftereffects. Our findings provide preliminary evidence to indicate that microstructural integrity of fibers directly damaged by stroke, and not the overall volume that remains, predicts the effect of noninvasive neuromodulation mediated downstream in the spinal cord.

## Introduction

Muscle weakness impairing voluntary control of the distal limbs after brain or spinal cord injury is a clinical manifestation of damage to the corticospinal tract [1]. Impairment results from a disruption of signaling along the cranial or spinal course of the tract onto spinal motor neuron pools [2]. Activity-based therapy via high-volume task repetition is thought to promote formation of new synapses and/or alter connectivity of existing synapses to support limb control [3]. However, rates of recovering distal limb functions compromised by damage to the brain and spinal cord remain low [4]. Both invasive and noninvasive stimulation techniques have been used as part of neuromodulation strategies that transiently increase movement potential of muscles weakened by neurological injury. When used in combination with activity-based therapy, these neuromodulation strategies are thought to leverage the nervous system's adaptive capacity more readily than task repetition alone [5].

Paired corticospinal-motoneuronal stimulation (PCMS) is a noninvasive neuromodulation strategy that involves pairing pre- and post-synaptic depolarization events in the spinal cord [6]. Activation of cortical motor neurons in primary motor cortex (M1) via single-pulse transcranial magnetic stimulation (TMS) depolarizes presynaptic terminals, and antidromic activation of spinal motor neurons via transcutaneous electrical stimulation of a peripheral nerve depolarizes postsynaptic terminals. As part of an original investigation in neurologically-intact controls, PCMS increased the size of motor-evoked potentials (MEPs) elicited by electrical stimulation of the cervicomedullary junction and voluntary motor output during brief, ballistic contractions of the elbow flexors [7]. Later studies showed similar effects in muscles of the hand [8] and ankle [9] in humans with spinal cord injury (SCI). Subsequent work has demonstrated an enhancement of outcomes in response to activity-based therapy after SCI [10,11], increased recruitment of spinal motor neurons in stroke survivors [12], and an acceleration of motor learning in controls [13,14]. There is growing evidence to support the therapeutic potential of PCMS. However, individual differences in the response to this neuromodulation strategy have been documented [9,13,15], and the physiological basis underlying variation in the clinical populations that might benefit remains largely unknown.

Alterations in cortico-spinal connectivity resulting from PCMS are thought to be mediated in synapses linking cortical and spinal motor neurons via principles of spike timing-dependent plasticity (STDP) [16]. The ability to elicit STDP-like changes in synaptic efficacy by way of PCMS would appear to have some reliance on the extent to which descending volleys elicited by single-pulse TMS propagate through damaged portions of the tract, irrespective of whether the damage exists in its cranial (e.g., stroke) or spinal (e.g., SCI) course. This consideration is particularly relevant in the months to years after onset of neurological insult when white matter has stabilized and the tract has remodeled. In our studies of stroke survivors with longstanding hand impairment, for instance, we have observed considerable loss of white matter volume in the residual corticofugal projection from M1 [17]. In many cases, however, cortico-spinal transmission is preserved, and minimal sparing of the tract does not appear to preclude the possibility of retaining voluntary control of the digits [18]. Relatedly, we previously observed that the magnitude of unrestricted water diffusion caudal to portions of the tract directly damaged by stroke predicts voluntary and stimulation-evoked activation of spinal motor neuron pools innervating paretic hand muscles [19]. It is therefore unclear whether and where along the residual tract the overall amount of surviving white matter or its microstructural integrity explain variation in the response to PCMS.

Here, stroke survivors (n ​= ​20) with longstanding hand impairment underwent PCMS, and M1 corticofugal projections were reconstructed in a subset (n ​= ​16) not contraindicated to MRI. We hypothesized that white matter within the region directly damaged by stroke, without specificity to its macro- or microstructural properties, would account for between-subject variation in MEP facilitation following PCMS.

## Materials and Methods

### Subjects

Characteristics of individuals (n ​= ​20) with hand impairment due to first-ever cerebrovascular accident (62.5 ​± ​10.6 years of age, 8.6 ​± ​6.5 years post stroke onset, 10 female) who underwent testing are presented in Table 1. Radiological reports from the medical record were accessed to verify stroke type and location at the time of onset. Subjects were screened for contraindication to MRI or noninvasive stimulation and excluded as appropriate. Subjects with musculoskeletal conditions affecting the arms/hands or any other condition that would prevent them from following instructions were also excluded. Each subject provided signed informed consent to undergo research procedures approved by the Institutional Review Board at VA Pittsburgh Healthcare System in accordance with guidelines established by the Declaration of Helsinki.

**Table 1:** Characteristics of stroke survivors.

ID	Age	Sex	Stroke type	Chronicity (months)	Side affected	MVC (N)	MEPMAX (mV)	Overlap volume (%)	PCMS|SHAM
1	58	M	R Basal Ganglia	116	ND (L)	26.3	0.472	38.7	Y|Y
2	58	M	Unknown	186	ND (L)	11.3	0.313	–	Y|Y
3	63	M	R MCA	75	ND (L)	29.5	0.225	44.4	Y|N
4	70	M	L Capsule	44	D (R)	9.3	0.133	81.7	Y|Y
5	71	F	R MCA	259	ND (L)	50.4	0.484	60.2	Y|Y
6	72	M	L MCA	63	D (R)	58.6	0.706	51.3	Y|Y
7	72	M	L MCA	99	D (R)	55.9	0.497	–	Y|Y
8	61	F	L Capsule	55	D (R)	19.1	0.129	20.5	Y|Y
9	69	M	Unknown	29	ND (R)	47.4	1.153	–	Y|Y
10	74	M	L MCA	240	D (R)	53.2	0.684	60.6	Y|Y
11	54	F	Unknown	21	D (R)	29.9	0.843	–	Y|Y
12	59	M	R MCA	119	ND (L)	34.2	0.065	30.9	Y|Y
13	72	F	L Capsule	50	ND (R)	26.0	0.117	49.9	Y|N
14	66	F	R Capsule	194	D (L)	23.9	2.067	20.7	Y|N
15	61	M	L MCA	17	D (R)	47.3	1.177	70.7	Y|N
16	25	F	L MCA	208	D (R)	21.2	0.191	18.8	Y|N
17	59	F	L MCA	133	D (R)	5.6	0.304	33.5	Y|N
18	60	F	L Capsule	113	D (R)	6.4	0.104	53.4	Y|N
19	61	F	R Pontine	23	D (L)	24.7	0.157	2.9	Y|N
20	64	F	R MCA	11	ND (L)	13.3	0.433	51.3	Y|N

M, male; F, female; R, right; L, left; MCA, middle cerebral artery; ND, non-dominant; D, dominant; MVC, maximal voluntary contraction; Y, yes; N, no.

### Diffusion MRI protocol

Subjects underwent MRI using a Prisma Fit 3T (Siemens, Erlangen, Germany) with a 64-channel head/neck receiver radio-frequency (RF) coil. Cushions were placed on either side of the head within the coil cage to restrict movement and to ensure bicommissural alignment. Structural MRI was acquired using T1 magnetization-prepared rapid acquisition with gradient-echo (MPRAGE) and T2 sampling perfection with application-optimized contrasts using a different flip angle evolutions (SPACE) sequence with the following parameters: repletion time (TR)/inversion time (TI)/echo time (TE) ​= ​1900/900/1.67 ​ms, voxel resolution ​= ​1.3-mm isotropic, acceleration factor ​= ​2, acquisition time ​= ​3 ​min 32 ​s; and TR/TE ​= ​3200/412 ​ms, voxel resolution ​= ​1-mm isotropic, acceleration factor ​= ​2, acquisition time ​= ​3 ​min 49 ​s, respectively). Diffusion spectrum imaging (DSI) data were acquired via a diffusion-weighted single-shot, twice-refocused, two-dimensional, multi-slice spin echo planar imaging (EPI) sequence (TR/TE ​= ​2480/99.2 ​ms, voxel resolution ​= ​2-mm isotropic, multi-band factor ​= ​4, partial Fourier factor in phase encoding (PE) ​= ​6/8, 258 diffusion directions with *b* values of 4000 ​s/mm^2^ and one with a *b*-value of 0 in the anterior-to-posterior direction; acquisition time ​= ​11 ​min). A separate sequence with a *b*-value of 0 was acquired (posterior-to-anterior PE direction, acquisition time ​= ​19 ​s) to correct for spatial distortion in the diffusion weighted MR image.

### PCMS protocol

At the outset of testing, subjects sat in a chair with the forearm maintained in a neutral position on an arm tray and held a 6-axis force sensor (Mini40, ATI Industrial Automation, Apex, North Carolina, USA) between the index finger and thumb with remaining digits fully flexed. Force signals were sampled at 200 ​Hz using custom software written in MATLAB version 2021a (The MathWorks Inc., Natick, Massachusetts, USA). The noise floor of the sensor was defined by sampling data while it was positioned on a stable surface. Subjects then grasped the sensor and were verbally exhorted by the experimenter to produce a true maximum voluntary contraction (MVC) for 5 ​s while the force signal was recorded. Two MVCs were obtained from each subject. An attempt was repeated if the subject dropped the sensor or reported losing grip.

Once MVCs were obtained, skin overlying the first dorsal interosseous (FDI) was prepared with an abrasive cream and cleansed with alcohol prior to securing surface electrodes (Ag–AgCl, 10 ​mm diameter) in a muscle belly-tendon montage. EMG signals were amplified, band-pass filtered (200–2000 ​Hz), and sampled at 2 ​kHz (Power1401, Signal, Cambridge Electronic Design Ltd., Cambridge, UK). Then, subjects were provided with instruction on subsequent procedures involving noninvasive stimulation. Single-pulse TMS does not elicit MEPs consistently in all stroke survivors, and MEPs elicited by TMS are influenced by certain factors (e.g., position of the limb, subject attention, background muscle activity, etc.) that require consideration. Multiple steps were taken to this end in efforts to preserve data fidelity and maximize the potential for future studies to reproduce findings reported here [20]. First, the experimenter assisted each subject identify a comfortable posture for the paretic hand/arm that they could maintain throughout the protocol, particularly those with increased muscle tone. Second, the experimenter instructed the subject on how to allow the surface supporting their paretic limb (e.g., arm tray or subject's lap) bear its entire weight to ensure background muscle activity was minimized. Subjects who exhibited spontaneous motor unit discharges were given real-time visual feedback of the EMG signal as an additional guide. Third, after inspecting the real-time signal to verify hand muscles were fully relaxed, the experimenter gave the same instruction at all time points to fixate gaze and hold attention stable prior test stimulation. Fourth, subjects were instructed to count the number of times they heard the stimulator discharge during conditioning stimulation, and the experimenter inquired on their count five times throughout to ensure attention was maintained. Fifth, subjects were asked to prioritize scheduling each session at a time of day when they felt most alert.

Single monophasic, magnetic pulses intended to elicit posterior-to-anterior currents in M1 were applied to the scalp with a Magstim 200^2^ stimulator (The Magstim Company Ltd., Whitland, UK) through a figure-of-eight coil (D70, 70-mm loop diameter). The 10–20 system was used to establish scalp locations corresponding to the vertex (Cz) and 7 ​cm lateral of Cz in line with the tragus of the ear contralateral to the target muscle. The optimal scalp site was determined by administering TMS pulses 7 ​cm lateral of Cz and moving the coil in ∼1-cm increments in anterior-posterior/medial-lateral directions. The coil was rotated to identify the optimal angle relative to the mid-sagittal plan. The coil location and orientation that produced stable MEPs at the lowest possible stimulator output was set as the optimal site and registered in a frameless, stereotaxic neuronavigation system (Brainsight, Rogue Research Inc., Quebec, Canada) used to ensure coil location and orientation was maintained throughout the entire protocol.

Next, resting motor threshold (RMT) was established as the minimal stimulator output needed to produce MEPs that were >50 ​μV in amplitude from 5 of 10 TMS pulses. Then, MEP_MAX_ was established by administering 5 TMS pulses at stimulator outputs just below and above RMT in 5-increment steps until maximum stimulator output was reached. If background muscle activity became persistently elevated or spontaneous motor unit discharges were observed, then another MEP_MAX_ was obtained. In some stroke survivors, MEPs could be elicited only by using maximal or near-maximal stimulator output, in which case steps for obtaining RMT and MEP_MAX_ were abbreviated. After establishing both, one set of 20 TMS pulses (0.25 ​Hz) was administered with the FDI muscle in a pre-contracted state. Real-time feedback of the rectified, smoothed EMG signal was displayed, and the subject was instructed to maintain sufficient muscle tension to hold the signal on a horizontal line corresponding to ∼10–30 ​% of their maximum. At baseline, 2 sets of 10–20 TMS pulses (0.25 ​Hz) were administered using a stimulator output that elicited MEPs with a mean peak-to-peak amplitude corresponding to approximately 50 ​% of MEP_MAX_ [[8], [9], [10], [11],^13^,^15^], which is the point on the recruitment curve where slope tends to peak and reflects the most efficient neuronal recruitment by TMS. For subjects in whom MEPs could be elicited only by using maximal or near-maximal stimulator output, a total of 10 TMS pulses were administered per set without a target amplitude.

MEP latencies from pre-contracted and resting muscle states were identified while subjects were given a brief break in preparation for maintaining the posture of their arm/hand throughout the remainder of the protocol. Bipolar felt pad electrodes soaked in a saline solution were secured to skin overlying the ulnar nerve at the wrist just proximal to the ulnar styloid with the cathode and anode separated by 2-cm and the cathode positioned opposite the FDI muscle. Foam cushions were placed under the hand and forearm to elevate both from the surface supporting both, thereby, preventing changes in pressure applied to the stimulating electrode. Electrical current (200 μs pulse duration) was supplied by a constant-current stimulator (DS7R, Digitimer Ltd., Welwyn Garden City, UK). Current was graded higher until the peak-to-peak amplitude of the M-wave saturated then decreased in small increments to establish M_MAX_. A total of 40 pulses were administered (1 ​Hz) with current amplitude set at M_MAX_ threshold to antidromically activate spinal motor neurons and elicit F-waves. M-and F-wave latencies were identified, and all response latencies were entered into equations to calculate central (CCT) and peripheral conduction times (PCT) as follows:

$$\text{CCT}={\text{MEP}}_{\text{Latency}}\;–\;\left(\text{PCT}+{\text{M-wave}}_{\text{Latency}}\right)$$

$$\text{PCT}=\left({\text{F-wave}}_{\text{Latency}}\;–\;{\text{M-wave}}_{\text{Latency}}\right)\;*\;0.5$$

For conditioning stimulation, devices were triggered (0.1 ​Hz) so that descending volleys arrived in the C8-T1 spinal cord ∼3 ​ms before antidromic volleys. A total of 120 stimulus pairs were administered over the course of 20 ​min. TMS output was set at 1.5xRMT (or maximum), and current amplitude was set at M_MAX_ threshold. In a subset of subjects who underwent the SHAM protocol (n ​= ​11), the TMS coil was placed on its side over the optimal site to produce the same pressure on the scalp and the same sound from the Magstim 200^2^ device as in the PCMS protocol; all remaining procedures were identical. Subjects were re-registered in the neuronavigation system as necessary to account for any shifts in the tracker affixed to the forehead. Test stimulation (i.e., 10–20 pulses per time point) was administered on a fixed time course following the final pair of conditioning stimuli at 10 ​min, 20 ​min, and 30 ​min using the same stimulator output from prior to conditioning stimulation that elicited a mean peak-to-peak amplitude of ∼50 ​% MEP_MAX_ at baseline. The number of time points and pulses for test stimulation was selected in keeping with our prior work [13] and to account for the Magstim 200^2^ device potentially disabling toward the end of conditioning stimulation due to high RMTs in some stroke survivors from our cohort.

### DSI data processing

**Fig. 1:**
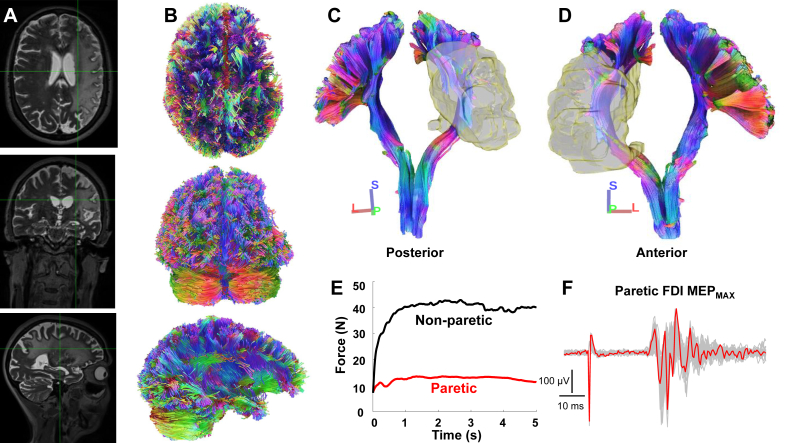
**A)** T2 anatomical image and **B)** whole brain tractography from axial (top), coronal (middle), and sagittal (bottom) views in a stroke survivor (ID 20 in Table 1). Coronal **C)** posterior and **D)** anterior view of M1 corticofugal projections (gray mask corresponds to the abnormal region). Note the extent of white matter loss and overlap with the tract. **E)** Maximal precision grip force from the non-paretic (black trace) and paretic (red trace) hand. **F)** MEP_MAX_ from the paretic FDI muscle (red trace) with all MEP waveforms (gray traces) overlaid.

DSI preprocessing was completed using FSL (https://www.fmrib.ox.ac.uk/fsl) and included motion correction, eddy correction, and co-registration between T1, T2, and MNI152 standard brain images with the three-dimensional DSI image. Fig. 1 contains a T2 anatomical image (Fig. 1A) and corresponding views of whole brain tractography (Fig. 1B) from a representative stroke survivor. DSI studio software (https://dsi-studio.labsolver.org) was used to visualize fibers from intact and residual tracts. A deterministic fiber tracking algorithm (angular threshold ​= ​60°, step size ​= ​1 ​mm) was used to reconstruct M1 corticofugal projections in both hemispheres to the caudal end of the image volume at the level of the brainstem. Fibers were generated using the co-registered precentral gyrus (i.e., M1) template from the Harvard-Oxford Cortical Structural Atlas and the Corticospinal Tract template from HCP842 Tractography Atlas as separate regions of interest. Tracts segmented by two independent raters were merged prior to quantifying directional diffusivities (eigenvalues, λ1 ​> ​λ2 ​> ​λ3) of each axial slice from M1 to the brainstem.

Masks corresponding to areas of abnormality were manually drawn on T1-and T2-anatomical images, which were then co-registered to the DSI image (Fig. 1C&D). As noted above, we frequently observe considerable atrophy of brain tissue in individuals with longstanding strokes, making it difficult to quantify the relative composition of lesioned parenchyma versus voids filled with cerebrospinal fluid. We therefore define the portion of the corticofugal projection falling within the volume of the abnormal region as the *overlap compartment*, and the portion below this level as the *caudal compartment*. Both fractional anisotropy (FA) and mean diffusivity (MD) were computed as the average from all slices in each compartment. White matter volume contained within the overlap compartment expressed as a percentage of white matter volume within the entire residual tract was used to compute *overlap volume*, which is reported for each stroke survivor in Table 1. White matter volume in a region analogous to the overlap compartment within the intact cerebral hemisphere was quantified and defined as the *mirror* compartment. Volumes from overlap and mirror compartments were used to compute *proportional volume*, which is the ratio of white matter volume in each of these compartments normalized to volumes of entire residual and intact tracts.

### Force & EMG data processing

Force signals recorded while subjects generated MVCs were analyzed using MATLAB version 2021a (Fig. 1E). Maximal precision grip force was taken as the mean value calculated from 2 ​s centered around the peak force. MEPs elicited by single-pulse TMS (i.e., test stimulation) were inspected to verify stability of the EMG signal 100 ​ms prior to onset of a given pulse. MEPs were removed from the analysis if peak-to-peak amplitude of the signal within this window was ≥2 standard deviations above the mean across all time points. Mean MEP size was computed at each test stimulation time point. MEP_MAX_ was taken as the mean of 5 MEPs with the highest peak-to-peak amplitude (Fig. 1F), and mean MEP values at each time point were normalized to MEP_MAX_. Normalized baseline MEP size was subtracted from normalized MEP size at each time point following conditioning stimulation, and the mean change across time points was computed.

### Statistical analyses

Statistical tests were completed using SPSS version 23 (SPSS Inc., Chicago, IL, USA) with significance set at p ​< ​0.05 a priori. Repeated-measures analysis of variance (ANOVA) was used to test for differences in normalized MEP size across time points in the sample of subjects who underwent the PCMS protocol (n ​= ​20) and the subsample who underwent both the PCMS protocol and diffusion MRI (n ​= ​16). Data from the subsample (n ​= ​11) who underwent testing in PCMS and SHAM conditions were entered into a 4 (time point) x 2 (condition) repeated-measures ANOVA.

Interrater reliability was established for fiber number within tracts segmented by independent raters via calculation of intraclass correlation coefficients (ICC) using 95 ​% confidence intervals (CI) based on absolute-agreement, two-way mixed-effects models. Paired-samples t-tests were used to test for differences in white matter volume between residual and intact tracts as well as between overlap and mirror compartments. Two-way repeated-measures multivariate analysis of variance (MANOVA) was used to test for main effects of tract (residual, intact) and compartment (overlap, caudal) on FA and MD.

Stepwise linear regression was used to determine the relative contribution of white matter macrostructure and microstructure in predicting mean MEP facilitation following PCMS. Predictors included FA and MD in both overlap and caudal compartments as well as proportional volume. These variables were included based on previously noted findings [19] and to minimize redundancy that exists between MD and other DSI metrics (i.e., axial and radial diffusivities) in regression models. A separate model including these same variables was used to predict maximal precision grip force. Collinearity diagnostics and plots of residuals were inspected to verify multicollinearity and homoscedasticity assumptions for linear regression were met. Criteria for variable entry and removal were set at a probability of F-to-enter ≤.05 and a probability of F-to-remove ≥0.10. For all statistical analyses, the Shapiro-Wilk test was used to verify that data distributions were approximately normal across all levels of each independent variable, Mauchly's test of sphericity was used to verify homogeneity of variance, and Greenhouse-Geisser correction statistics were used when sphericity could not be assumed. The Bonferroni test was used to correct for multiple comparisons.

## Results

**Fig. 2:**
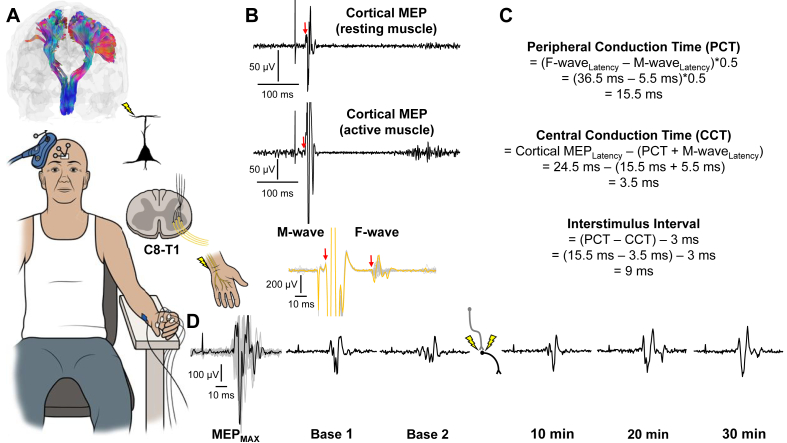
**A)** Coronal view of M1 corticofugal projections with representation of subject during conditioning stimulation and each neural element depolarized with noninvasive stimulation shown to the right (ID 10 in ​Table 1). **B)** Electrophysiological response latencies (red arrows) were entered into **C)** equations to calculate peripheral and central conduction times for determining when to trigger stimulation devices. **D)** MEP_MAX_ and MEP waveforms at each test stimulation time point before and after conditioning stimulation.

**Fig. 3:**
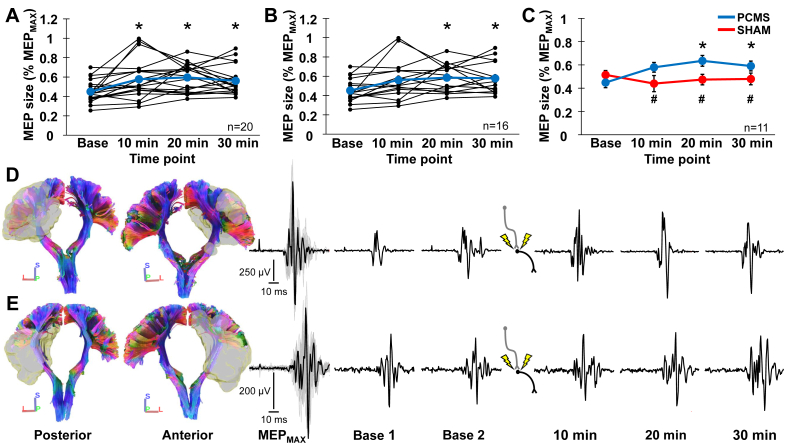
Line plots showing MEP size normalized to MEP_MAX_ across time points for **A)** the sample (n ​= ​20) who underwent the PCMS protocol and **B)** the subsample (n ​= ​16) who underwent the PCMS protocol and diffusion MRI. Black lines in both plots correspond to individual subjects, and blue lines correspond to the sample mean. **C)** Line plot showing MEP size normalized to MEP_MAX_ across time points for the subsample (n ​= ​11) who underwent both PCMS (blue line) and SHAM (red line) protocols. Coronal posterior (left) and anterior (right) views of M1 corticofugal projections in stroke survivors who **D)** did (ID 15 in Table 1) and **E)** did not (ID 6 in Table 1) show MEP facilitation in response to the PCMS protocol. Corresponding MEP_MAX_ and MEP waveforms at each test stimulation time point before and after conditioning stimulation are shown to the right. Difference relative to baseline (∗p ​< ​0.05) or between conditions (^#^p ​< ​0.05).

Fig. 2 shows M1 corticofugal projections (Fig. 2A), response latencies (Fig. 2B), conduction time calculations (Fig. 2C), and electrophysiological waveforms (Fig. 2D) from a stroke survivor who underwent the PCMS protocol and diffusion MRI. MEP size normalized to MEP_MAX_ was 45 ​± ​11.2 ​% at baseline, 57.8 ​± ​20.1 ​% at 10 ​min, 59.6 ​± ​14.2 ​% at 20 ​min, and 56.1 ​± ​14.4 ​% at 30 ​min, on average, in subjects who underwent the PCMS protocol. In this sample (n ​= ​20), a facilitation in MEP size was detected (F_(3, 57)_ ​= ​7.683, p ​< ​0.001, $\eta_{p}^{2}$ ​= ​0.29) at 10 ​min (p ​= ​0.022), 20 ​min (p ​< ​0.001), and 30 ​min (p ​= ​0.006) relative to baseline (Fig. 3A). MEP size normalized to MEP_MAX_ was 45.3 ​± ​12.5 ​% at baseline, 56.1 ​± ​20.1 ​% at 10 ​min, 58.6 ​± ​14.3 ​% at 20 ​min, and 57.9 ​± ​15.6 ​% at 30 ​min, on average, in subjects who underwent the PCMS protocol and diffusion MRI. In this subsample (n ​= ​16), a facilitation in MEP size was detected (F_(3, 45)_ ​= ​5.685, p ​= ​0.002, $\eta_{p}^{2}$ ​= ​0.28) at 20 ​min (p ​= ​0.005) and 30 ​min (p ​= ​0.015) relative to baseline (Fig. 3B). A time point ​× ​condition interaction (F_(3, 30)_ ​= ​5.834, p ​= ​0.003, $\eta_{p}^{2}$ ​= ​0.37) was detected in the subsample (n ​= ​11) tested under PCMS and SHAM protocols. MEP size normalized to MEP_MAX_ was greater in the PCMS condition relative to the SHAM condition at 10 ​min (p ​= ​0.046), 20 ​min (p ​= ​0.009), and 30 ​min (0.003) (Fig. 3C). PCMS facilitated MEP size at 20 ​min (p ​= ​0.001) and 30 ​min (p ​= ​0.028) relative to baseline, whereas MEP size was unchanged by the SHAM protocol across time points. Fig. 3 shows M1 corticofugal projections and corresponding MEP waveforms for stroke survivors who did (Fig. 3D) and did not (Fig. 3E) show MEP facilitation in response to the PCMS protocol. Taken together, these results indicate that PCMS had the intended effect of increasing cortico-spinal connectivity at all or most time points, whereas, the SHAM protocol had no such effect.

**Fig. 4:**
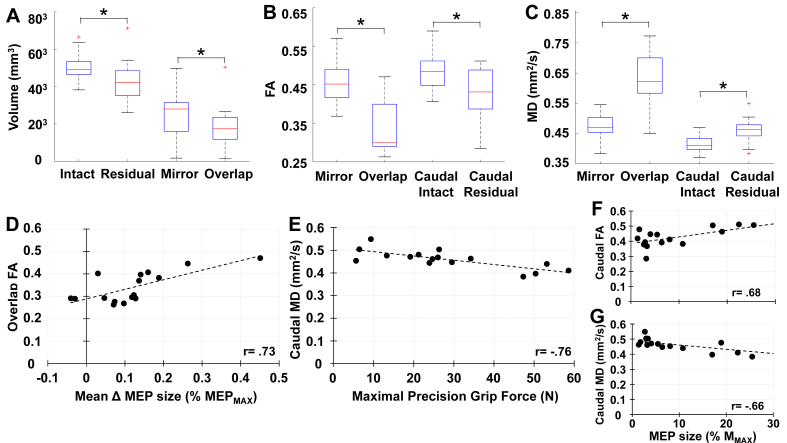
Box plots showing comparison of **A)** white matter volume, **B)** FA, and **C)** MD by tract and compartment (∗p ​< ​0.05). Scatterplots showing significant prediction of **D)** the mean change in MEP size by FA in the overlap compartment, **E)** maximal precision grip force by MD in the caudal compartment, **F)** MEP_MAX_ by FA in the caudal compartment, and **G)** MEP_MAX_ by MD in the caudal compartment.

ICCs established a high degree of agreement between raters on fiber numbers for both residual (ICC ​= ​0.979, 95 ​% CI ​= ​0.934–0.993) and intact (ICC ​= ​0.985, 95 ​% CI ​= ​0.958–0.995) tracts. Slices within overlap and caudal compartments comprised 42.7 ​± ​8.3 ​% and 32.4 ​± ​12.1 ​% of the overall tract, respectively. Asymmetries in white matter volume were detected between sides, with reductions observed in the residual tract (*t*_(15)_ ​= ​3.13, p ​= ​0.003) and overlap compartment (*t*_(15)_ ​= ​3.08, p ​= ​0.004) (Fig. 4A). A tract ​× ​compartment interaction was detected (*Wilks’* Λ ​= ​0.32, F_(2,14)_ ​= ​14.573, p ​< ​0.001, $\eta_{p}^{2}$ ​= ​0.676) for both FA (F_(1, 15)_ ​= ​10.721, p ​= ​0.005, $\eta_{p}^{2}$ ​= ​0.417, Fig. 4B) and MD (F_(1,15)_ ​= ​29.457, p ​< ​0.001, $\eta_{p}^{2}$ ​= ​0.663, Fig. 4C). FA was reduced within overlap ($\bar{D}$ ​= ​-0.116, $s_{\bar{D}}$ ​= ​0.019, p ​< ​0.001) and caudal ($\bar{D}$ ​= ​-0.05, $s_{\bar{D}}$ ​= ​0.021, p ​= ​0.031) compartments, whereas MD was increased within overlap ($\bar{D}$ ​= ​0.158, $s_{\bar{D}}$ ​= ​0.022, p ​< ​0.001) and caudal ($\bar{D}$ ​= ​0.044, $s_{\bar{D}}$ ​= ​0.01, p ​< ​0.001) compartments on the residual side. These results indicate an overall reduction of white matter volume on the residual side and within the abnormal region, as well as diminished microstructural integrity within and below the abnormal region on the residual side.

Stepwise linear regression produced a single model, with FA in the overlap compartment significantly predicting the mean change in MEP size following PCMS (F_(1, 14)_ ​= ​15.52, p ​= ​0.001, *R*^*2*^ ​= ​0.526, Adjusted *R*^*2*^ ​= ​0.492). An *increase* in the fraction of directionally-dependent diffusion within the overlap compartment was associated with an *increase* in MEP size (*t* ​= ​3.94, p ​= ​0.001, *β* ​= ​0.725, Fig. 4D). Pearson product-moment correlations demonstrated that positive trends between the mean change in MEP size following PCMS and the ratio of entire tract volumes (r = 0.298, p = 0.263) or proportional volume (r = 0.388, p = 0.137) were not statistically significant, indicating that the amount of white matter surviving injury was weakly associated with PCMS aftereffects. The mean change in MEP size was uncorrelated with MEP_MAX_ (*r* ​= ​0.057) and maximal precision grip force (*r* ​= ​0.073), indicating that stimulation-evoked and voluntary activation of spinal motor neuron pools innervating paretic hand muscles were unrelated to PCMS aftereffects.

Consistent with our prior work [19], stepwise linear regression containing the same five variables resulted in a single model, with MD in the caudal compartment significantly predicting maximal precision grip force (F_(1, 14)_ ​= ​19.14, p ​< ​0.001, *R*^*2*^ ​= ​0.578, Adjusted *R*^*2*^ ​= ​0.547). An *increase* in the magnitude of unrestricted diffusion in the caudal compartment was associated with a *decrease* in force-generating capacity (*t* ​= ​−4.38, p ​= ​0.001, *β* ​= ​−0.76, Fig. 4E). Pearson product-moment correlations showed that a positive trend between maximal precision grip force and MEP_MAX_ was not statistically significant (r ​= 0.436, p ​= ​0.091) and that maximal precision grip force was not associated with the ratio of entire tract white matter volume (r ​= ​−.260, p ​= ​0.332) or proportional volume (r ​= ​−0.272, p ​= ​0.308). MEP_MAX_ was significantly correlated with FA (r ​= ​0.676, p ​= ​0.004, Fig. 4F) and MD (r ​= ​−0.656, p ​= ​0.007, Fig. 4G) in the caudal compartment, but its association in the overlap compartment was weak and not statistically significant for FA (r ​= ​.399, p ​= ​0.125) and did not exist for MD (r ​= ​−0.155).

## Discussion

The purpose of this study was to examine properties of white matter macrostructure and microstructure underlying inter-individual variation in the response to a noninvasive neuromodulation strategy previously shown to increase cortico-spinal connectivity through STDP-like mechanisms in the spinal cord. PCMS upregulated connectivity of the residual tract in our cohort of individuals with longstanding hand impairment secondary to stroke. Regression analyses revealed that the fraction of directionally-dependent diffusion along residual fibers within the region directly damaged by stroke predicted MEP facilitation following the neuromodulation protocol. Despite asymmetries in white matter volume within this region and the entire residual tract, macrostructure was unrelated to the effect of PCMS. This effect, therefore, appears to have more to do with integrity of the residual tract and less to do with how much of it remains. Findings from the current study are relevant to therapeutic applications that seek to resolve paresis following damage to descending tracts mediating voluntary limb control.

Single-pulse TMS elicits MEPs recorded from skeletal muscle via *trans*-synaptic activation of pyramidal cells in M1 that, in turn, discharge a series of descending volleys which summate on spinal motor neuron pools [21]. The precise mechanism by which a magnetic pulse applied to the scalp activates M1 varies according to certain parameters (e.g., intensity [22], coil orientation [23], pulse waveform [24], etc.), which were held constant throughout the PCMS protocol. We theorized that gains in cortico-spinal transmission mediated by PCMS have some general reliance on how well volleys propagate through damaged portions of the tract. Analyses revealed that microstructural integrity in the overlap compartment explained approximately half of the variance in MEP facilitation relative to baseline. Although MEP size has been associated with distal limb impairment after stroke [25,26], the positive trend between MEP_MAX_ and maximal precision grip force observed here did not reach statistical significance. There also was no trend between MEP_MAX_ and the response to PCMS. Principles that apply to stimulation-evoked recruitment of spinal motor neuron pools also apply to voluntary recruitment [27]. We used maximal precision grip force as a surrogate of voluntary recruitment and found that it also did not trend with the response to PCMS. While microstructural integrity of fibers within the region directly damaged by stroke appears to have a strong contribution to eliciting changes in synaptic efficacy via STDP-like principles downstream in the spinal cord, the effect appears unrelated to how well descending volleys originating at the level of cortex depolarize spinal motor neuron pools.

A decrease in the fraction of directionally-dependent diffusion (i.e., FA) and increase in the overall magnitude of unrestricted diffusion (i.e., MD) are typically observed in the chronic stage of stroke [28], which is attributable to a breakdown of myelin and disintegration of axons that reduce barriers to diffusion. This inverse pattern is not always observed in the caudal compartment several months after stroke onset [29] but was detected in both overlap and caudal compartments in the cohort of stroke survivors studied here. There tends to be a greater change in microstructural properties of white matter directly affected by stroke than in the perilesional space at the chronic stage [30], which might explain why FA within only the overlap compartment predicted MEP facilitation in response to PCMS despite evidence of pathological changes in white matter microstructure within both compartments. It is plausible that fiber microstructure in the overlap compartment might be in some way related to broader network compensation, making this portion of the tract more or less responsive to neuromodulation. There is also the possibility that the integrity of reciprocal connections within the tract (i.e., ascending and descending fibers) contributes to the effect of PCMS in some way, which may be particularly relevant in individuals with neurological injury who exhibit diminished inhibitory control at one or more levels of the neuraxis. To this end, we have observed responses elicited by single-pulse TMS that appear to comport with activation of transcortical reflex loops in some stroke survivors [18]. We also have studied portions of the tract rostral to the overlap compartment previously [19] but did not observe any association between microstructural properties in this rostral compartment with voluntary or stimulation-evoked activation of paretic hand muscles. There was no trend observed with MEP facilitation in the current study, suggesting that the integrity of fibers in closest proximity to a magnetic field that falls off rapidly as a function of the distance from the TMS coil does not account for the effect of PCMS.

Secondary (i.e., Wallerian) degeneration of white matter is associated with distal limb impairment in chronic stroke survivors [31]. An asymmetry in FA has been observed in portions of the tract at the level of the brainstem by the chronic stage [32,33], but this asymmetry is not always apparent [19] and can be absent [34] when the comparison includes the entire compartment caudal to the portion directly damaged by stroke. In contrast to mean MEP facilitation following PCMS and some prior work [25], both maximal precision grip force and MEP_MAX_ were not significantly correlated with fiber microstructure in the overlap compartment but strongly associated with microstructure in the caudal compartment. The caudal compartment contains brainstem structures, giving rise to descending motor pathways that are phylogenetically older and thought to compensate for damage to the corticospinal tract further upstream [35,36]. The cortico-reticulo-spinal system, for instance, contributes to gross aspects of limb control (e.g., force generation) after stroke [37]. Fibers converging on the brainstem origin of these alternate pathways overlap in cortical origin and intermingle throughout their cranial course [38]. Corticofugal projections studied here were restricted to M1. However, the contribution of alternate descending pathways to coarser aspects of limb control may explain why residual tract microstructure in the caudal compartment was strongly linked to voluntary and stimulation-evoked activation of spinal motor neuron pools innervating paretic hand muscles.

The extent of overlap between the lesion and residual tract is associated with motor impairment at the chronic stage of stroke [[39], [40], [41]] and predicts recovery of the same [[42], [43], [44]]. Although an asymmetry in white matter volume was detected within this compartment and the entire corticofugal projection from M1, proportional volume was only weakly associated with MEP facilitation in response to PCMS and not a significant predictor in the final regression model. The lack of an association would appear to signify a positive outlook for similar neuromodulation strategies, given potential for considerable loss of white matter within the residual tract long after stroke onset [17] and also because activity-based therapy in combination with noninvasive stimulation at the chronic stage has been shown to drive adaptions in residual white matter microstructure that support gains in paretic limb control [45]. Although the sample studied here included cases with severe structural damage, further work is needed to understand upper limits in this regard.

Few human studies exist on white matter adaptation along the spinal course of descending motor pathways after stroke. From a methodological standpoint, low signal-to-noise ratio at the level of the brainstem and spinal cord limits spatial resolution, complicating the ability to partition and isolate different tracts within the cord. Nevertheless, the limited available evidence indicates that white matter microstructure at the cervical level is linked to distal limb impairment [46,47]. Greater awareness of adaptation in microstructural properties of white matter along the spinal course of the residual tract can inform therapeutic tools that aim to increase the gain of input onto spinal motor neurons [12,48], which adapt to the loss of trophic input yet remain morphometrically intact after stroke [see [49] for a review].

Findings of the current study provide preliminary evidence of white matter physiology underlying the response to an emerging noninvasive neuromodulation strategy late after stroke onset when disability tends to persist. Due to the limited sample size of this study, further work in a larger cohort of stroke survivors that also allows for inclusion of additional predictors is needed to gain further insight into the physiological profiles that might benefit from this particular form of neuromodulation. The MEP elicited by single-pulse TMS was the outcome measure used here to evaluate the response to PCMS. This electrophysiological parameter was practical for addressing the theoretical basis of our research question and also tends to be more tolerable than other stimulation techniques used to isolate the locus of adaptation, which the preponderance of evidence supports is mediated below the level of cortex and above the level of spinal motor neuron pools [[7], [8], [9],^12^,^15^,^50^,^51^]. The MEP elicited by single-pulse TMS is a strong prognostic indicator of motor recovery after stroke [52], and we have shown MEP facilitation in response to PCMS is a predictor of changes in force control [13]. However, behavioral gains resulting from administering PCMS in combination with activity-based therapy might be considered the superlative therapeutic currency of this noninvasive neuromodulation strategy. Further work is needed to evaluate clinically meaningful effects and to determine whether the same properties and regions of residual tract microstructure that predict MEP facilitation also apply to gains in movement capabilities of the paretic limb.

## Author contributions

**Michael A. Urbin**: Conceptualization, Methodology, Software, Validation, Formal analysis, Investigation, Resources, Data curation, Writing, Visualization, Supervision, Project administration & Funding acquisition.

**Fang Liu**: Methodology, Software, Validation, Formal analysis, Data curation, Writing & Visualization.

**Chan Hong Moon**: Methodology, Software, Investigation & Visualization.

## Declaration of competing interest

The authors declare the following financial interests/personal relationships which may be considered as potential competing interests: Michael A. Urbin reports financial support was provided by US Department of Veterans Affairs. If there are other authors, they declare that they have no known competing financial interests or personal relationships that could have appeared to influence the work reported in this paper.
